# Fracture resistance of endodontically treated teeth restored with resin post reinforced with glass fiber

**DOI:** 10.25122/jml-2020-0180

**Published:** 2021

**Authors:** Mihaela Chirila, Bogdan Dimitriu, Ruxandra Ioana Bartok, Oana Amza, Ana Maria Serban, Ioana Suciu

**Affiliations:** 1. Department of Endodontics, Faculty of Dental Medicine, “Carol Davila” University of Medicine and Pharmacy, Bucharest, Romania; 2. Department of Esthetics, Faculty of Dental Medicine, “Carol Davila” University of Medicine and Pharmacy, Bucharest, Romania; 3. Private dental practice, Bucharest, Romania

**Keywords:** fractures resistance, resin post, glass fiber, FRC

## Abstract

This study aims to determine whether the design of resin posts reinforced with glass fiber (FRC) and Reporfost (Angelus, Londrina, PR, Brazil) significantly improves the fracture resistance of endodontically treated teeth restored through this method.A batch of 30 maxillary monoradicular teeth (15 central incisors, 15 canines) were treated endodontically by step-back technique (apical enlargement 40-K file) sealed with Sealapex (Kerr Corporation, Orange, US) and gutta-percha by lateral condensation, cold. They were divided into two equal groups, prepared for cementing the FRC posts. The Exacto posts (Angelus, Londrina, PR, Brazil) in group 1 and the Reforpost posts (Angelus; Londrina; PR, Brazil) were cemented with dual cure resin cement Breeze Self-Adhesive Resin Cement (Pentron Clinical, Orange, US). Fracture resistance testing was performed on the crown-apical axial direction, using the Hounsfield / Tinius Olsen H1-KS, PA, USA mechanical testing apparatus. The behavior of each tooth-post assembly was recorded as a graph. The statistical analysis was done using one way ANOVA (α=0.05). The differences between the Exacto post group and the Reforpost post group are not statistically significant (p = 0.466). The maximum force recorded was 970 N and the minimum 186N. The mean force at which the fracture occurred was approximately 500N for both groups. The strain test showed that modifying the Reforpost post design did not improve the fracture resistance parameters of the tooth-post assembly through increasing the surface friction or maintaining adhesion to the walls of the root dentin.

## Introduction

The amount of remaining hard structure determines the method of restoration after endodontic treatment decisively. The loss of dental tissue through extensive carious processes and also following the endodontic treatment itself, especially by configuring the access cavity, substantially changes the biodynamics of the endodontically treated tooth. The consequence of these changes predisposes the tooth to fracture, and if the retention of future restoration is uncertain, it is recommended to use a device with intraradical aggregation, namely a post.

In daily dental practice, the posts made of various materials: metal, zirconium, carbon, composite resins reinforced with glass fibers have been used for a long time. Posts made of carbon fiber, despite the resistance and elastic behavior similar to dentin, were gradually abandoned due to the non-physiognomic appearance. Practitioners turned their attention to more transparent posts reinforced with quartz, glass, or silicon-zirconium fibers [1]. In the case of an endodontically treated tooth, the role of a post is to facilitate the retention of the prosthetic restoration and to minimize the risk of fracture of the tooth under the action of occlusal forces [2]. Paradoxically, the preparation of the canal in order to insert a pivot weakens the root, and its manufacture from rigid materials (metal) increases the risk of fracture.

This aspect was solved by the appearance of glass fiber-reinforced composite (FRC) resin posts. The FRC positions manage to harmonize to some extent the discrepancies between the modulus of elasticity of the positions (approximately 18 GPa) with the modulus of elasticity of the dentin (approximately 20 GPa). This mechanical property contributes to reducing the risk of fracture by contributing to the distribution of stress in the root dentin in a relatively uniform manner [3].

An FRC layer contains the matrix polymer (epoxy resins or dimethacrylate-based resins) and glass fibers [4]. The adhesion of glass fibers to the polymer matrix depends on the type of resin used, which forms a continuous phase around the fibers, impregnating them. The mechanical properties of FRC are strength and rigidity and are conferred by glass fibers, which must be intact and unfragmented for optimal performance.

Although they do not effectively strengthen the structure of the teeth in which they are cemented, the composite resin posts reinforced with glass fibers can generate a more homogeneous distribution of stresses within the assembly, reducing the risk of root fracture. Several dimensional factors, including the length and thickness of the post, but also clinical factors such as the amount of hard dental structure and the degree of adaptation of the post to the dentinal substrate, are vital for the strength of the restoration. The correct selection of positions in accordance with the clinical situation and a meticulous clinical procedure is essential for obtaining a successful restoration [4, 5].

The posts with parallel faces are more retentive than the conical ones and achieve a more uniform distribution of stress in functionality along the entire length. The taper of the post must be consistent with the taper of the preparation. Taper influences pivot retention: the higher the taper, the lower the retention [5].

FRC posts are retained on the dentinal wall by means of adhesion with cement. There are passive positions, and as a result, accurate adaptation to the root wall is very important. The FRC shape, which can be cylindrical, conical, or combined, confers different performance parameters in terms of stress retention and distribution. The cylindrical posts have a very good retention and evenly distribute the stress along the entire length of the canal. The conical posts offer sufficient retention, with the preservation of the dentin in the coronary portion of the canal [6].

Since in the case of metal stations it was found that the active ones have better retention compared to the passively cemented metal stations, it was natural to try to improve the retention of the FRC pivots by modifying the design.

With a wide range of prefabricated FRCs and various adhesive types of cement, the restoration of endodontically treated teeth is still in search of the safest and most efficient technical solution. To select appropriate protocols for clinical use, mechanical tests that simulate intraoral conditions are important tools for evaluating possible scenarios [7].

This in vitro study aims to analyze the influence of changing the design of resin posts reinforced with glass fibers on their retention under certain conditions of functional stress.

## Material and Methods

A group of 30 monoradicular teeth (15 central maxillary incisors and 15 maxillary canines) with a closed apex were included in the study. The specimens were immersed in a 0.5% Chloramine solution for 48 hours. They were subsequently examined visually and radiographically to exclude teeth with possible cracks, fractures, internal resorptions, and root curvatures greater than 10°. The set of teeth was kept until the experiment was performed in distilled water.

The access cavities were created with a crack cutter (SS White, Lakewood, NJ, USA). The canal and working length were determined using Kerr 10 needles. Root canal shaping was performed with manual needles (K-files; Dentsply-Maillefer, Ballaigues, Switzerland) using step-back technique using 0.02 taper needles up to size 40 ISO up to the apex, and those ISO 45, 50, 55 were used by shortening the working length by 1 mm successively after each needle.

### Post endodontic restoration

The root canals were unclogged by removing with Gates Glidden cutters and heated instruments on a predetermined length, keeping 3 mm apical from the canal filling. The preparation of the canal for the pivot was done with Peeso cutters corresponding to the diameter of the pivot. Pivots no. 1 Exacto (Angelus) and no. 2 were used (Reforpost, Angelus).

Before cementing, the pivots were cleaned with alcohol and dried. Breeze self-adhesive resin cement (Pentron Clinical, Orange, USA) was used for cementation.

Breeze self-adhesive resin cement is easy to use and comes with a self-mixing system. The cement was applied according to the manufacturer's instructions; the pivot was inserted under digital pressure for 10 seconds, and the excess was removed and photopolymerized for 40 seconds. After cementing the pivot, a matrix was adapted, and the tooth was reconstructed directly with Filtek Z250 composite (3M, St. Paul, MN, USA).

### Fracture resistance test

**Figure 1.**
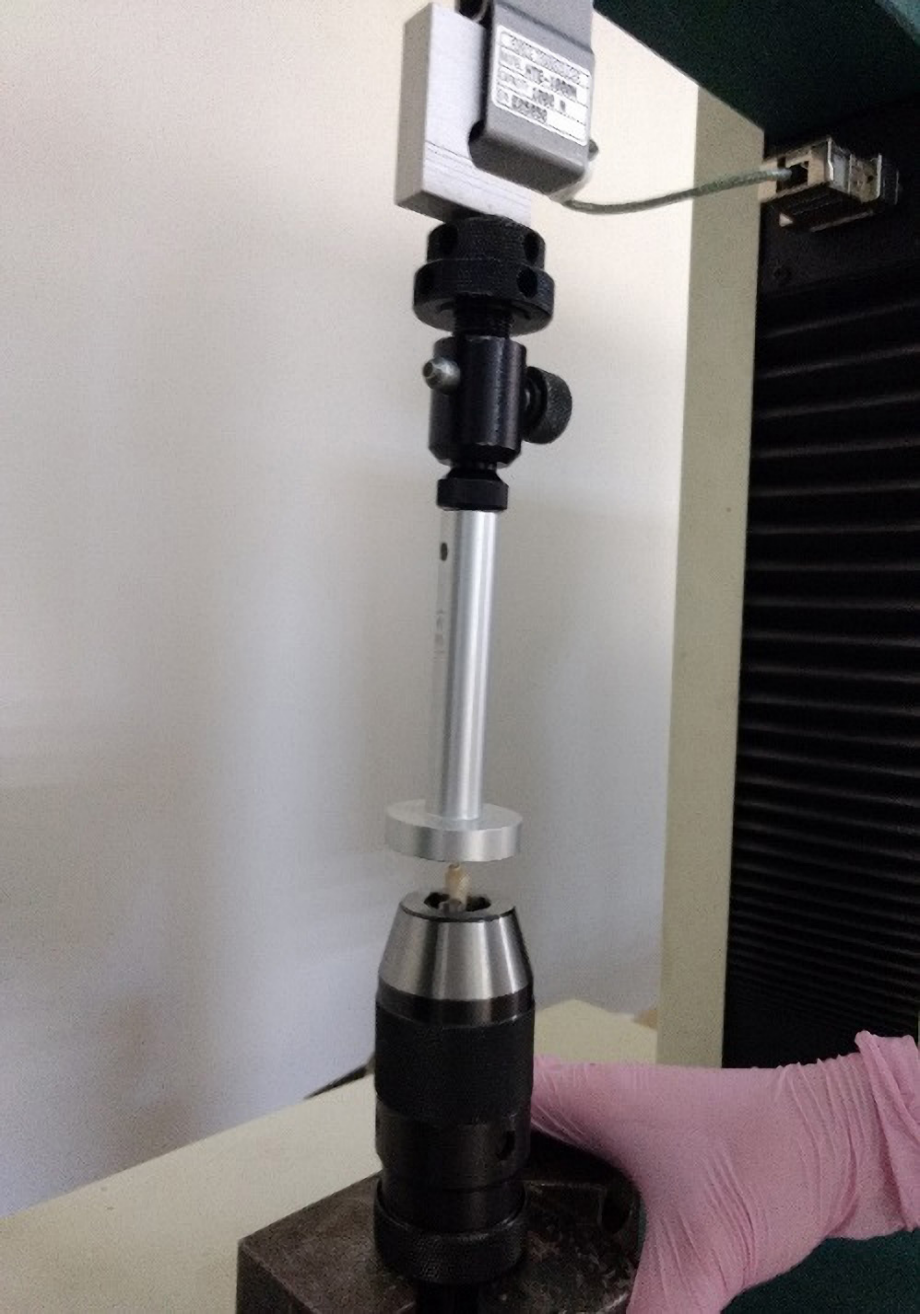
Tooth fixed in unit for mechanical tests using the Hounsfield/Tiniuus Olsen H1KS tester

Testing the fracture strength of post-tooth units was performed using the Hounsfield/Tinius Olsen H1-KS mechanical tester (Figure 1). For this, a load cell with a capacity of 1000 Newtons was used. Each tooth was tested in turn, being fixed in a mandrel provided with three guide rods. The chuck was placed on a fixed flat plate to support the test; then, the device was controlled using the operating panel and QMAT software, causing the uniaxial displacement of the load cell of 1000 Newtons (with a preset speed of 1 mm/minute) to the tooth fixed in the mandrel, compressing it with the help of the pressing head. 

The teeth were fixed and subjected to a static compressive load in the longitudinal axis at a crosshead speed of 1 mm/minute using the Hounsfield/Tinius Olsen unit for mechanical tests (H1KS model) until there was a sudden drop in the stress-strain curve. The load levels at the fracture point were measured.

### Statistical analysis 

The statistical analysis was done using one-way ANOVA (α=0.05). Between the Exacto post and the Reforpost populations, the differences were not statistically significant (p=0.466215). Therefore, the null hypothesis stating that the design differences of the Reforpost devices improve the fracture resistance of the post-tooth assembly was rejected.

## Results

**Figure 2.**
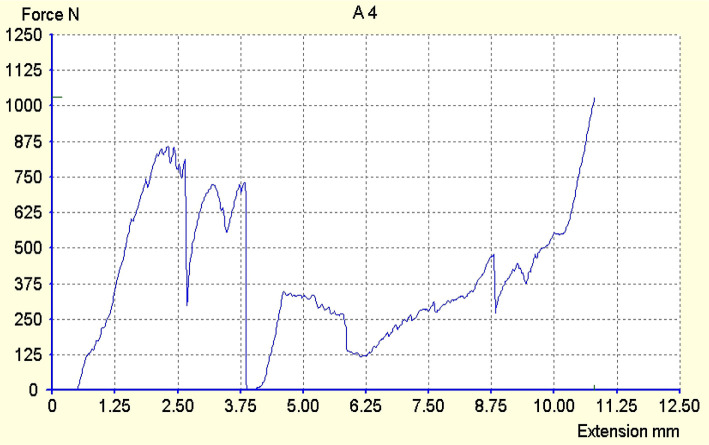
Registration of deformations of the tooth 1.1 restored with Exacto post.

**Figure 3.**
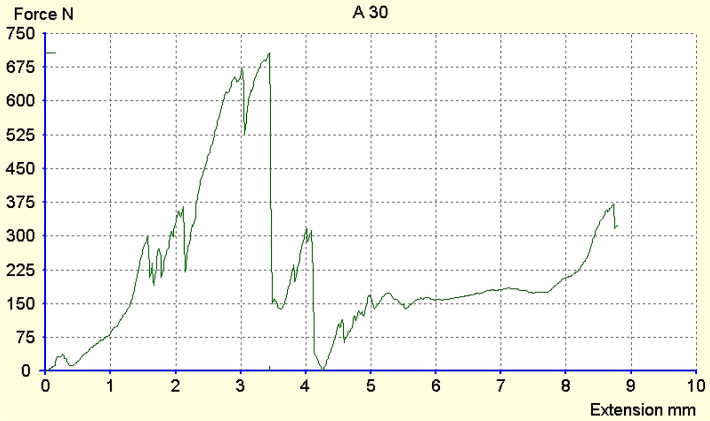
Registration of deformations of the tooth 1.3 restored with Exacto post.

**Figure 4.**
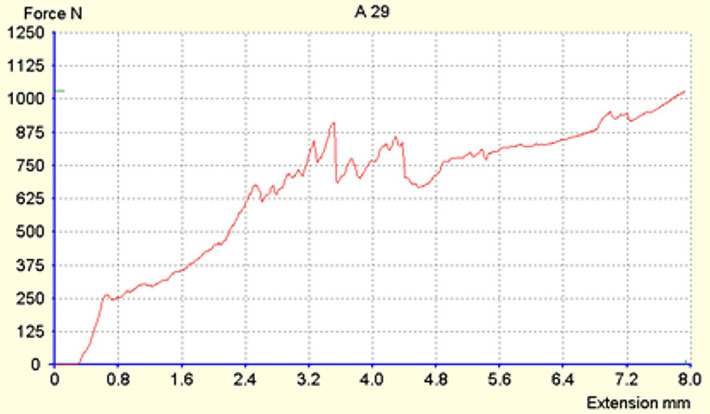
Registration of deformations of the tooth 1.3 restored with Reforpost post that remained intact.

The maximum force registered was 970N (Figures 2, 3), and the minimum was 186N. The average force at which a fracture occurred was approximately 500N for both populations. From the Exacto group, 4 teeth remained intact (Figure 3) after running the test, while from the Reforpost group, only one tooth remained intact (Figure 4).

**Figure 5.**
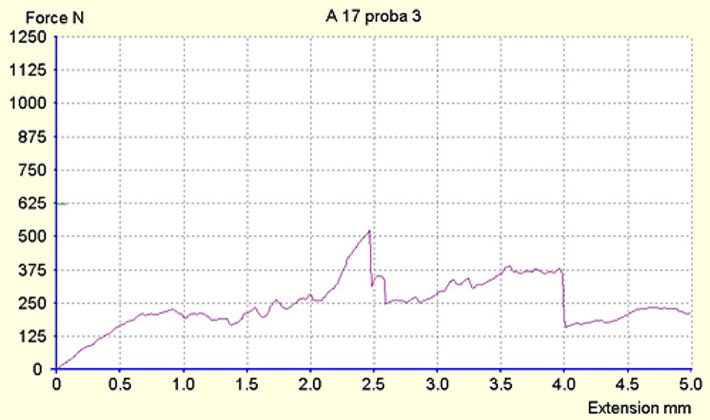
Registration of deformations of the tooth 2.3 restored with Reforpost pivot that remained intact.

Looking at the tooth classes, the average resistance of canine teeth was approximately equal between the two populations tested, at 524.26N (Figure 5). For the central incisor teeth, the average resistance was recorded to be 474.26N.

The fracture of the post-tooth occurred in an oblique coronal-apical direction and affected the interface between the tooth and the cement for both groups tested.

## Discussion

This study analyzed the behavior of the front teeth restored with posts produced by Exacto or Reforpost (Angelus, Londrina, PR, Brazil) under the action of a force exerted vertically in the axis of the teeth.

**Figure 6.**
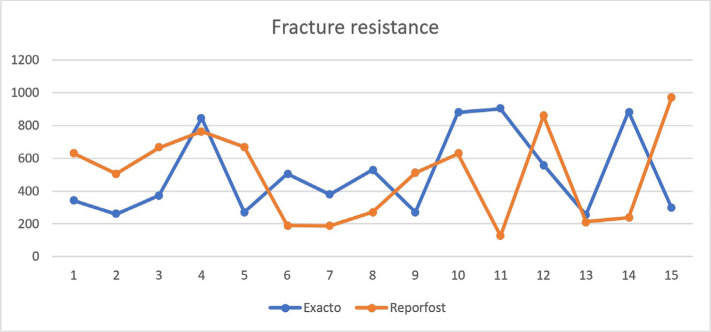
Fracture resistance of endodontically treated teeth restored with resin post reinforced with Exacto and Reforpost glass fiber (Angelus, Londrina, PR, Brazil).

Extreme values were recorded in the Reforpost group. The maximum force recorded was 970 N, and the minimum was 186N (Figure 6). The mean force at which the fracture occurred was approximately 500N for both groups.

The composition of the two types of posts is similar, with a content of 80% glass fibers and 19–20% epoxy resin. From the Exacto group, 4 teeth remained intact at a force of 880N, and from the Reforpost group, 1 tooth remained at the value of 970N.

In both groups of teeth, the average resistance of the canines was approximately equal. In the Reforpost group, it was 476.625N, while for Exacto it was 578.71N, with a total average of 524.26N. For the central incisors, the average resistance registered in the Exacto group was 438.12N, and for Reforpost was 515.57N, with a total average of 474.26N. This aspect is partially justified by the diameter of the posts used for each group. In the Exacto group, the post used was no. 1, while no. 2 was used in the Reforpost group; the most accurate adaptation to the canal walls was respected for all the teeth included in the study.

It is accepted that the increase in the diameter of the post causes an increase in fracture strength due to the distribution of stress over a larger area. The resistance to occlusal loading is higher if a post with a greater diameter is used [8].

There is a correlation between the mechanical properties and the structural characteristics of the posts as in the composition of the posts. These characteristics include the integrity, size, density, and distribution of the fibers, as well as the nature of the bond between the matrix and the fibers, which may be the determining factors for different flexural strength values [9].

Novais *et al.* determined that the glass fiber ratio is 68% for the Exacto posts (Ø = 1.45mm) and 59% for the Reforpost posts (Ø = 1.43). Flexural strength is 835.9 MPa for Exacto and 569.5 MPa for Reforpost.

The post’s fastening system typically forms a strong adhesion between the core and the tooth tissue: a single hollow unit, a post-cement mono-block in which the loading forces are evenly distributed to all mono-block components is created. This mono-block is a complex system composed of several parts that operate multi-axial stress fracture forces, unevenly distributed, which depend on the external load and its direction. When a tooth is exposed to stress, the glass threads stretch evenly and join to their breaking point [10].

Usually, the fibers play the role of load-bearing member, while the resinous matrix maintains the fiber in the desired location and orientation, effectively acting as a means of load transfer between them and protects them from environmental damage. Thus, the mechanical properties are dependent on the stability of the interface region between the matrix surfaces and the fibers [11].

The use of resin cement significantly increases the retention of fiber posts and improves the fracture resistance of glued structures to withstand functional forces better, improving marginal adaptation with better apical sealing, increases the retention of posts with a short length, and optimizing fracture patterns if they appear [2].

The bonding properties of self-adhesive resin cement are based on functional acid methacrylate, which can simultaneously demineralize and infiltrate tooth substrates, potentially being able to chemically bind to hydroxyapatite [2]. In our study, we preferred the self-adhesive cement, dual-cure Breeze Self-Adhesive Resin Cement (Pentron) to speed up this process because the goal was focused on studying the potential improvement of strength due to the design of overlapping cones of Reforpost posts.

Reforpost had the lowest flexural strength; a possible explanation for this could be the weak link of the interface caused by irregularities and bubbles produced during the manufacturing process. Such discontinuities along the interfaces between the matrix and the fibers are evidence that the strength of the interfacial link is critical [9].

In the case of Reforpost posts, obtaining the design of overlapping cones led to the reduction of the number of fibers/surface area and the appearance of manufacturing defects with negative consequences on the physical properties [9], confirmed in the present study.

Mechanical tests that simulate intraoral conditions are important tools for evaluating restorative materials and techniques, thus contributing to selecting certain protocols for clinical use.

## Conclusion

The fracture resistance of the teeth endodontically treated and restored with FRC posts, respectively Exacto and Reforpost (Angelus), did not register statistically significant differences. The load test in this study, with its obvious limitations, showed that changing the design of Reforpost posts did not improve the fracture resistance of the tooth-post assembly by increasing the adhesion surface or retentivity at the root dentin walls. Our study suggests that the adhesion between the epoxy resin of the studied posts and the cementing cement is not mechanically influenced by the post surface design.

## Acknowledgments

### Conflict of interest

The authors declare that there is no conflict of interest.
